# Stimulus-Response-Outcome Coding in the Pigeon Nidopallium Caudolaterale

**DOI:** 10.1371/journal.pone.0057407

**Published:** 2013-02-20

**Authors:** Sarah Starosta, Onur Güntürkün, Maik C. Stüttgen

**Affiliations:** Department of Biopsychology, Faculty of Psychology, University of Bochum, Bochum, Germany; Bowling Green State Universtiy, United States of America

## Abstract

A prerequisite for adaptive goal-directed behavior is that animals constantly evaluate action outcomes and relate them to both their antecedent behavior and to stimuli predictive of reward or non-reward. Here, we investigate whether single neurons in the avian nidopallium caudolaterale (NCL), a multimodal associative forebrain structure and a presumed analogue of mammalian prefrontal cortex, represent information useful for goal-directed behavior. We subjected pigeons to a go-nogo task, in which responding to one visual stimulus (S+) was partially reinforced, responding to another stimulus (S–) was punished, and responding to test stimuli from the same physical dimension (spatial frequency) was inconsequential. The birds responded most intensely to S+, and their response rates decreased monotonically as stimuli became progressively dissimilar to S+; thereby, response rates provided a behavioral index of reward expectancy. We found that many NCL neurons' responses were modulated in the stimulus discrimination phase, the outcome phase, or both. A substantial fraction of neurons increased firing for cues predicting non-reward or decreased firing for cues predicting reward. Interestingly, the same neurons also responded when reward was expected but not delivered, and could thus provide a negative reward prediction error or, alternatively, signal negative value. In addition, many cells showed motor-related response modulation. In summary, NCL neurons represent information about the reward value of specific stimuli, instrumental actions as well as action outcomes, and therefore provide signals useful for adaptive behavior in dynamically changing environments.

## Introduction

Pigeons are classic model animals for the study of learning and choice, and psychological research employing pigeons as subjects forms the backbone of contemporary learning theory [1]–[7]. Although the wealth of available behavioral and neuroanatomical data renders the pigeon a highly suitable model system for behavioral neuroscientists too, few studies so far investigated single-neuron responses in pigeons during operant behavior. Here, we examined the response properties of single neurons in the nidopallium caudolaterale (NCL) while the birds performed a perceptual decision task.

The NCL is a multimodal associative forebrain area that receives input from secondary sensory areas of all modalities and projects to both limbic and sensorimotor striatum as well as premotor areas [8], [9]. NCL lesions impair executive processes such as working memory and reversal learning [10], [11] while leaving sensory discrimination and motor performance unaffected [12]. Converging evidence from neurochemical [13]–[15], anatomical [8], [16], behavioral [10], [17]–[19], and electrophysiological [20], [21] studies point to functional equivalence of NCL and mammalian prefrontal cortex that possibly results from a process of convergent evolution [9], [22].

Like many neurons in prefrontal cortex, NCL neurons fire in response to visual cues predictive of reward as well as to (water) reward itself [20], [23]. To date, all of the few available single-unit recording studies have examined NCL neurons under experimental conditions where anteceding cues were easily discriminable, unambiguously identified the correct response to be made, and thus fully predicted whether the trial would end in reward. In contrast, natural environments are inherently uncertain in terms of decision-relevant sensory information and action outcomes, as external stimuli convey only probabilistic information about upcoming rewards [24]–[26], and such uncertainty about action outcomes is reflected e.g. in orbitofrontal neurons [27], [28]. Another open issue is to what extent neurons firing in response to cues predicting reward (or non-reward) also fire when reward is presented (or omitted) and thereby could provide generalized positive or negative valuation signals such as posited in theoretical accounts of reinforcement learning [29], [30].

The purpose of the present experiment therefore was to investigate a) whether NCL neurons' firing rate scales in proportion to subjective reward expectancy, b) to what extent these neurons generalize across types of events (e.g. firing for both reward-predicting cues and rewarding outcomes) and c) if NCL neurons fire when expectations are violated, such as when a predicted reward fails to materialize. We designed a task which allows for the assessment of the subjective probability that a reward will occur for each of several stimuli. In addition, the task allowed us to assess the relation of neuronal activity and motor behavior (key pecking) during stimulus presentation. We find that NCL neurons indeed represent task-related variables, such as the spatial frequency of specific sample stimuli, current motor output, and occurrence or non-occurrence of reward. Interestingly, a substantial fraction of NCL neurons specifically responded to sample stimuli predicting non-reward; moreover, the same neurons also fired when a reward was expected but not delivered, and may thus provide a negative valuation signal that could subserve learning from negative consequences.

## Materials and Methods

### Subjects

Five homing pigeons (*Columba livia*), obtained from local breeders and raised in the institute's own aviary, served as subjects. Animals were housed individually in wire-mesh cages inside a colony room with a 12 h dark-light cycle (lights off at 8 p.m.). Water was available at all times; food was restricted to the period of daily testing on workdays, with additional free food available on weekends. During the experiment, the pigeons were maintained at 80–90% of their free-feeding weight. All subjects were experimentally naïve and treated according to the German guidelines for the care and use of animals in science. All procedures were approved by a national ethics committee of the State of North Rhine-Westphalia, Germany.

### Behavioral apparatus

The operant chamber measured 34 cm×34 cm×50 cm. The back wall of the chamber featured a single translucent response key (4 cm by 4 cm, bottom height from the floor 17 cm) which could be transilluminated by an LCD flat screen mounted against the back wall of the experimental chamber. Each effective key peck produced an audible feedback click. Food (grain) was provided by a food hopper located below the center key. The chamber was housed in a sound-attenuating shell, and white noise was provided at all times to mask extraneous sounds. Sample stimuli were sine wave gratings of varying spatial frequency (range: 2 to 64 cycles per display (cpd)). All stimuli had equal contrast. The display on the flat screen subtended 10 by 10 cm and the translucent response key was positioned about 4.5 cm from the screen. Assuming the animals' eyes were 5.5 cm from the key when viewing the stimuli [31], this amounts to a viewing angle of roughly 40°, and the stimuli ranged from 0.02 to 0.64 cycles per degree viewing angle. Because the exact spatial frequencies of the stimuli are not of central importance in this paper, they will for simplicity be given in cycles per display rather than cycles per degree. All hardware was controlled by custom-written Matlab code (The Mathworks, Natick, MA; [32].

### Procedure

**Figure 1:**
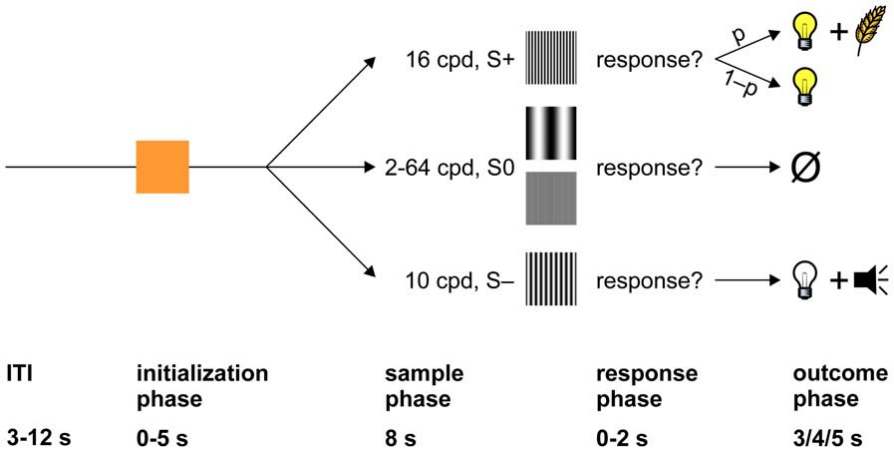
Illustration of the behavioral paradigm. After a variable ITI (3–12 s, truncated exponential distribution with a mean of 6 s), the response key was transilluminated orange (initialization phase). Following a response (a single key peck) within 5 s, one of several sample stimuli (sine wave gratings of varying spatial frequency) was presented for full 8 s (sample phase), plus 2 additional seconds or until the animal responded, whichever was shorter (response phase). If the animal did not respond within 2 s after the sample phase had elapsed, the screen turned blank, and a new trial ensued. If the animal responded, the trial's outcome depended on which stimulus was presented (outcome phase). If S+ was presented, a food hopper was illuminated and provided 3 or 4 seconds access to grain with probability p (reward); in the remaining trials, the food hopper was illuminated for the same time, but the hopper was not activated and, thus, no grain was available. If S– was presented, house lights were turned off, and a clearly audible tone was presented for 4 or 5 seconds (punishment). If S0 was presented, no consequence ensued.

Figure 1 illustrates the time course of individual trials of the behavioral paradigm. After a variable intertrial interval (ITI) whose duration was drawn from a truncated exponential distribution with a mean of 6 s (range: 3–12 s), the response key was transilluminated orange for up to 5 s (‘initialization phase’). If the pigeon did not respond with a single key peck within 5 s, the trial was terminated and the ITI started again. If the pigeon did respond to the initialization stimulus, the display was updated immediately to present one of several sample stimuli (sine wave gratings of varying spatial frequency). Each stimulus was presented for full 8 seconds regardless of the birds' behavior (‘sample phase’) and for another 2 seconds or until the animal responded (‘response phase’). If a response occurred during the latter phase, the response key went blank, and one of several possible consequences ensued (‘outcome phase’): if S+ (16 cpd) was presented, the food hopper was illuminated for 3 or 4 seconds (depending on the animal), and, provided food access during that interval with probability p, with p ranging from 0.6 to 0.8. On the other fraction of trials 1–p, the food hopper was illuminated for the same time but the food hopper was not activated (‘S+ food omission’). If the bird responded to S– (4 cpd in one session, 10 cpd in all others), all houselights were turned off for 5 s, and a clearly audible tone (sawtooth wave at 1000 Hz) was presented (punishment). If the bird responded to any of the other stimuli (denoted S0, spatial frequencies ranging from 2–64 cpd), the key turned blank but no other consequence ensued (‘S0 food omission’). Not responding to S– and failing to respond to S+ was inconsequential.

Even though responding during the sample phase was never reinforced, all animals pecked at the response key during that time window to some degree. The mean number of responses to each stimulus within that period was used to construct behavioral generalization gradients [33]. Although only responses to S+ were reinforced, the animals exhibited stimulus generalization as indexed by responding progressively less to stimuli as they became increasingly dissimilar from S+. Response rate can be taken as an index of reward expectancy: response rate increases monotonically with reinforcement rate [2], [34] and has been taken to index reward expectancy before [35], [36]. Honig [37] showed that generalization gradients can be used to predict preferences when stimuli are presented pairwise in a forced choice task. In this situation, animals consistently choose the stimulus which elicited the larger number of responses during the foregoing generalization test.

Stimuli were presented in pseudorandom sequence. Sessions contained between 300 and 510 trials (median: 400 trials). Altogether, we obtained 28 behavioral sessions with successful electrophysiological recordings. For three of the five birds (23 sessions), there were ten different gratings (eight S0s with 6, 12, 14, 18, 20, 24, 36, and 64 cpd). The remaining two birds (5 sessions) were tested with eight different gratings (six S0s with 2, 7, 20, 24, 32, and 45 cpd for one bird which contributed only one session, and 8, 13, 19, 22, 25, and 32 cpd for another bird which contributed four sessions). On average, each session contained 50 presentations of S+, 50 presentations of S–, and 24 presentations of each individual S0 (ranges were 23–77, 26–77, and 10–40, respectively). Sessions were conducted every other day and lasted about 130 minutes each.

### Surgery

After achieving stable performance, animals were implanted with custom-built movable microdrives [38], [39], each holding eight electrodes made from 25 µm formvar-coated nichrome wires (Stablohm 675; California Fine Wire, Grover Beach, USA) which were connected to microconnectors (Omnetics Connector Corporation, Minneapolis, USA). Pigeons were anesthetized with isoflurane, feathers on the skull were cut, and the animals were positioned in a stereotaxic apparatus. The skin overlying the skull was incised and pulled sideways. Five to six stainless steel microscrews (Small Parts, Logansports, USA) were placed on the skull for anchoring the dental cement head mount. One screw served as ground for electrophysiological recordings. A small trepanation was made in the skull overlying the left or right NCL. The location for implantation was chosen on the basis of stereotaxic coordinates of the NCL as described by [8]. The electrodes were targeted to the coordinates AP –5.5, ML ±7.0, and the microdrive was implanted such that the electrodes could be driven along the entire dorsoventral axis of the NCL. Light-curing dental cement was used to anchor the microdrive to the skull. Antibiotics were applied to the wound margins before the wound was sutured. Animals received analgesics (Carprofen, 10 mg/kg) for three days following surgery and were allowed to recover for a minimum of two weeks before testing.

### Electrophysiology

We recorded from six hemispheres in five birds (five left, one right). In each session, neuronal activity from seven microwires was recorded, the eighth microwire served as reference electrode. Electrodes were advanced at least 100 µm before each session. All units with sufficient signal-to-noise ratio were analyzed without preselecting for responsiveness. Signals were fed through a custom-built headstage with unity gain, amplified 1,000x and prefiltered online by a difference amplifier (DPA-2FS, npi electronic GmbH, Germany), and digitized using an analog-to-digital converter (power 1401 A/D system, Cambridge Electronic Design, Cambridge, UK) with a sampling rate of 16–20 kHz. The raw data was stored with Spike2 Version 7.06a (Cambridge Electronic Design) for offline processing. Prior to spike extraction, all channels were digitally bandpass-filtered from 500 to 5000 Hz. Spikes were detected with amplitude thresholds and were sorted manually using principal component analysis.

Sorting results were examined with custom-written Matlab code. Because previous studies examining single NCL neurons have failed to find evidence for spatial clustering of functionally similar neurons, and because extracellular unit recording is prone to record spikes from multiple non-separable units at a time [40], we chose to adopt very conservative criteria for classifying units as ‘single units’. To qualify as single unit, all of the following conditions had to be met: a) a clearly discernible cluster in principal component space, b) no evidence of overlapping multiple units both in waveform overlay and density plots [41], c) a unimodal, symmetrical distribution of peak waveform amplitudes without evidence of false negatives, d) absence of very short (<2 ms) interspike intervals, and a signal-to-noise ratio (SNR) of at least 2. SNR was calculated as the difference between the maximum and the minimum of the averaged waveform, divided by the range of the central 95% of data points in the noise distribution. Thus, assuming normally distributed noise, an SNR of 2 implies that the means of signal and noise distributions are separated by 8 standard deviations, implying that the distributions overlap by less than 0.01%. Units which did not meet all of the above criteria were marked as multi units and analyzed separately. However, the criterion of an SNR >2 held for both single and multi units. Mean SNR for single units was 4.0 (range 2.2–7.5). These criteria were deliberately set to be very conservative in order not to confound estimates of spontaneous firing rate and waveform width by inadvertent inclusion of multi units or single units with missed spikes.

To check for movement-related artifacts resulting e.g. from wing flapping or key pecking, all raw channels were inspected visually during and after recording, and channels with obvious artifacts were discarded. In addition, for each unit we examined the frequency distribution of spike counts relative to each registered key peck. All spike waveforms within ±20 ms of a key peck were plotted separately and compared to spike waveforms detected outside this window to ensure that the former were not pecking artifacts.

Spontaneous firing rate was calculated over the last three seconds before onset of the initialization phase. Spike count differences between trial phases were expressed as the area under the receiver operating characteristic curve (AUROC; [42], [43]. AUROC reveals how much information a neuron contains about which of two conditions are actually present to an ideal observer to whom only the total spike count is known. A value of 0.5 signifies complete overlap of the two distributions, while values of 0 or 1 denote complete separability of the two distributions. To facilitate interpretation, we rescaled AUROC such that a value of –1 implies perfect discriminability of conditions with stronger responding for non-reward, a value of +1 implies perfect discriminability of conditions with stronger responding for reward, and a value of 0 implies equal responding to both events (following [44]). In the manuscript, this measure is referred to as ‘outcome preference’. Because spike count distributions were heavily skewed (see results), we exclusively employed non-parametric hypothesis tests (Wilcoxon's rank sum test for 2 samples and Kruskal-Wallis test for >2 samples).

Spike-density functions (SDFs) were constructed by filtering peri-stimulus time histograms (PSTHs) with a Gaussian kernel with a standard deviation of 500 ms (all PSTHs) or 5 ms (PPTHs). The wide kernels for PSTHs were chosen because the large number of stimulus conditions as well as the very low firing rates would otherwise lead to cluttered visual displays. However, all statistical analyses were conducted using raw spike counts.

To determine the peaks and troughs of the spatial frequency tuning functions, we first normalized each neurometric gradient – mean firing rate as a function of log spatial frequency – such that values ranged from 0 to 1. Then, we fitted a normal distribution to the gradients. The fitting procedure used three free parameters – mean, standard deviation, and a factor controlling the height of the distribution. Means were constrained to lie between 0 and 100 cpd, and standard deviations could range from 0 to 12 cpd. Goodness of fit was assessed by r^2^. As a sanity check, we fitted the distributions to the psychometric gradients as well and obtained excellent fit qualities (all r^2^>0.91).

Units were examined for motor properties by comparing the spike count distribution within ±100 ms (split up into four time bins of 50 ms each) around all key pecks to a uniform distribution with the χ^2^ goodness-of-fit test. A prerequisite for this test was that each time bin contained at least 5 spikes. PPTHs were constructed only for key pecks that occurred at least 150 ms after the last registered key peck to exclude ‘double pecks’ that result from sequential upper and lower beak key contacts [45]. Joint stimulus- and motor modulation was assessed by comparing spike count distributions for separate stimuli by means of the Kruskal-Wallis-test. All analyses were done in MATLAB 7.8.0 (The Mathworks, Natick, USA).

### Reconstruction of recording sites

After completion of the experiments, pigeons were deeply anaesthetized with Equithesin (4.5–5.5 ml/kg body weight) and perfused intracardially with 0.9% saline (40°C) followed by 4% formaldehyde. Prior to anesthesia, 0.1 ml heparin was injected to prevent blood coagulation. Brains were embedded in gelatin before being sectioned at 40 µm. Every second slice was stained with cresyl violet. The point of largest expansion of the cannula track was used to estimate the position of the recordings sites along the anterior-posterior and the mediolateral axes.

## Results

### Behavior

**Figure 2:**
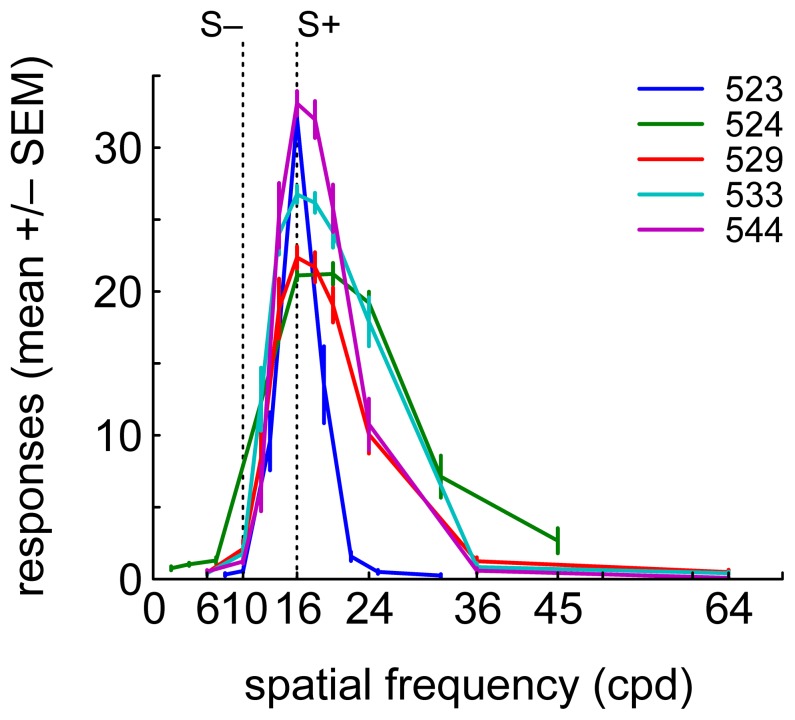
Behavioral data. Average psychometric generalization gradients for five birds, obtained by averaging the number of key pecks during the sample phase for each stimulus and each session for individual birds. S+ and S– are denoted by vertical dotted lines. Color-code identifies animal. Error bars denote SEM.

Animals reliably responded maximally to S+ and stimuli with similar spatial frequency and minimally or not at all to the stimuli with the lowest and highest spatial frequencies. The function relating the animals' average response rate to the sample stimuli will henceforth be referred to as psychometric generalization gradient. Figure 2 shows the five birds' averaged psychometric gradients. For individual animals, the gradients remained fundamentally unchanged across recording sessions, with a tendency to sharpen with experience. The fraction of S– trials in which the animals responded was consistently low: the median number of punishment trials across all physiological sessions was merely 3 (mean = 5). Therefore, neural responses to punishment were not analyzed.

### Basic electrophysiological properties of NCL neurons

**Figure 3:**
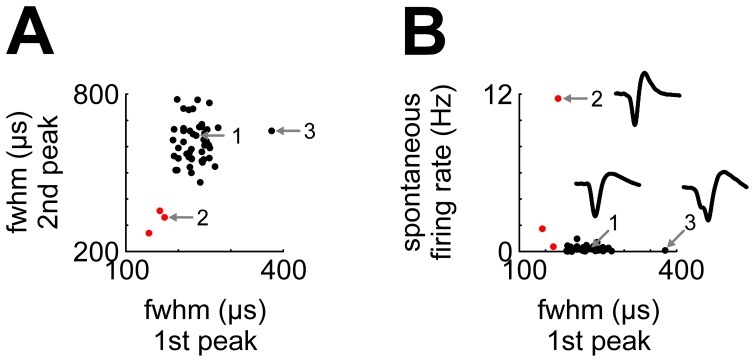
Single-unit waveform shape parameters. A) Plotting the width (full width at half maximum, fwhm) of the first spike phase against the width of the second spike phase reveals two discernible clusters (black and red). Red dots identify neurons with very short action potential durations; numbers 1–3 identify neurons whose average waveforms are shown in B). B) Neurons firing thin spikes have higher spontaneous activity. Conventions as in A.

Overall, we recorded 49 high-quality single neurons and 79 multi units from five birds. Spontaneous firing rates of NCL single neurons were extremely low (mean 0.47 Hz, median 0.18 Hz, range <0.01 to 11.7 Hz). During task events, average firing rates rarely exceeded 2 Hz, and even average peak firing rates almost never surpassed 5 Hz. Plotting the width of the first phase against the width of the second phase of the averaged waveforms revealed two discernible clusters of neurons (Figure 3A). The larger cluster (46/49 units, 94%; black) had peak widths ranging from 190 to 378 µs and 469 to 784 µs (full width at half maximum; first and second phase, respectively). The smaller cluster (3/49 units, 6%; red) had peak widths from 144 to 174 µs and 276 to 360 µs, thus classifying as “thin spikes” indicative of inhibitory interneurons (“Type III” neurons in [8]). These three neurons exhibited a considerably higher spontaneous firing rate (0.4, 1.7 and 11.7 Hz) compared to the other cluster of neurons (mean 0.2 Hz, ranging from <0.01 to 1 Hz; Figure 3B). In the following, we exclusively report data from single units; however, results were highly similar for multi units.

### Neural activity during the sample phase

#### Example neurons

**Figure 4:**
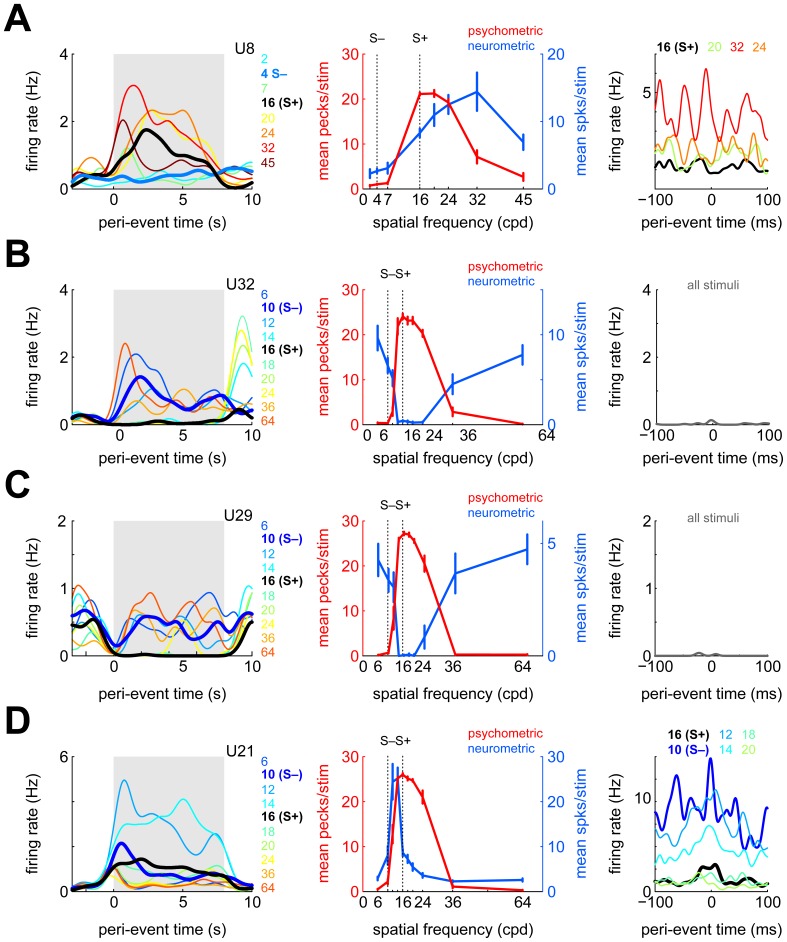
Response properties of four example neurons during the sample phase. A) Left panel: spike-density functions (SDFs) calculated during the sample phase, shown separately for each stimulus (color coded). Bold lines denote S+ and S–, respectively, gray shaded background highlights the duration of the sample phase. Middle panel: behavioral (psychometric, red) and neurometric (blue) generalization gradients. Error bars represent standard error of the mean (SEM), vertical dotted lines identify S+ and S–. Right panel: SDFs triggered relative to individual key pecks, split up according to which stimulus was present during key pecking (color-coded). B,C,D) As in A, but for three different neurons. For the neurons shown in B and C, there were too few spikes around key pecks split up the PPTH according to which stimulus was present.

Figure 4 shows four example neurons' activity during the sample phase. Figure 4A (left panel) depicts the activity of one neuron, split up and color-coded for different sample stimuli. Firing rates differed significantly across stimuli (χ^2^(9,365) = 133.3, p<10^−23^), with responding being significantly higher for S+ than S– (p<10^−11^, compare bold black and blue lines). However, the unit responded even more to some of the S0 stimuli than to S+, with maximal firing to 32 cpd. Accordingly, the neurometric generalization gradient (i.e., the mean spike count during the sample phase plotted separately for each stimulus; middle panel, blue line) appears as a shifted version of the behavioral generalization gradient (same panel, red line). Nonetheless, psychometric and neurometric gradients were positively correlated (r = 0.67, 95% confidence interval (CI95) −0.07 to 0.93). The correlation between psychometric and neurometric gradients will henceforth be referred to as ‘n-p correlation’.

A previous study suggested that a small fraction of NCL neurons carries premotor signals [20]. Therefore, it could be that the positive correlation between the behavioral and the neurometric gradients is due to increased firing during key pecking. However, this neuron did not exhibit significant motor modulation (χ^2^(3) = 2.6, p = 0.46). Also, splitting up the PPTH according to which stimulus was present at the time of key pecking (right panel) reveals that stimulus identity modulates average firing rates around key pecks in a manner consistent with the average neurometric gradient seen in the middle panel: firing rate during pecking on 32 cpd is highest, followed by 24, 20 and finally 16 cpd (χ^2^(3) = 12.5, p = 0.006). Thus, the modulation of firing rate during the sample phase is not due to motor-related activity but due to the sample stimuli (see below for more detailed analyses of motor-related modulation).

Figure 4B shows the activity of another NCL neuron whose firing rate was significantly stimulus-modulated during the sample phase (χ^2^(9,323) = 187.5, p<10^−34^). Unlike the previous example, this neuron responded considerably more to S– than to S+ (p<10^−13^); however, responses were strongest to the two S0 stimuli most dissimilar to S+ (6 and 64 cpd; left panel, dark blue and red lines, respectively). The neurometric generalization gradient was almost a perfect mirror image of the psychometric generalization gradient obtained in the same session (middle panel; r = −0.95, CI95 −0.99 to −0.80). The neuron exhibited little activity during the ITI (median spontaneous firing rate 0 Hz, mean 0.3 Hz) and during key pecking (right panel).

The neuron shown in Figure 4C also showed differential stimulus modulation during the sample phase (χ^2^(9,230) = 123.1, p<10^−21^, left panel) as well as a significantly negative n-p correlation (r = −0.98, CI95 −1.00 to −0.93, middle panel). Unlike the neuron in Figure 4B, the negative correlation was due to reduced responding to S+ and similar stimuli, rather than enhanced responding to extreme spatial frequencies. Similar to the previous example, this neuron hardly fired during key pecking (right panel).

Finally, Figure 4D shows the activity of a fourth NCL neuron whose firing rate was significantly modulated during the sample phase (χ^2^(7,277) = 99.3, p<10^−17^). The neuron fired almost exclusively for the two sample stimuli with spatial frequencies intermediate between S+ and S– (left panel). Accordingly, its n-p correlation was moderate and not significant (r = 0.36, CI95 −0.35 to 0.81, middle panel). The PPTH showed an obvious peak around the time of registered key pecks, indicative of its motor-related response modulation (right panel, black line; χ^2^(3) = 56.4, p<10^−11^). Splitting up the PPTH according to which sample stimulus was present during key pecking reveals that, on top of the observed motor modulation, this unit was modulated by the currently visible sample stimulus and could therefore be regarded as coding for a contextual action: key pecking when certain stimuli, but not others, are present (χ^2^(6) = 24.3, p = 0.0005).

#### Population response

**Figure 5:**
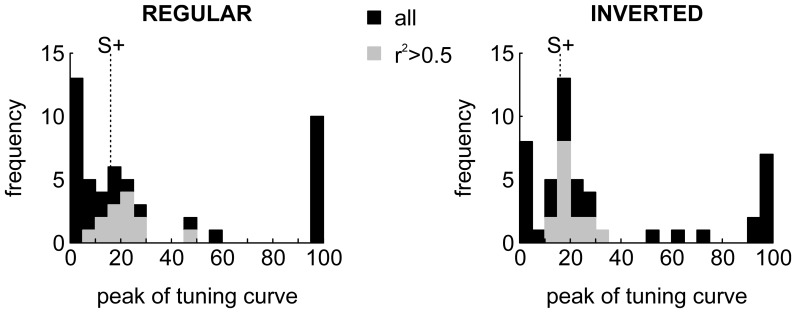
Distributions of tuning peaks for neurometric gradients. Left panel, peaks of Gaussian tuning functions fitted to the neurometric gradients. Black bars represent peaks for all neurons, gray peaks only for those neurons for which the goodness of fit exceed a value of r^2^ = 0.5. Right, same as left, but neural gradients were inverted before fitting Gaussian curves by first subtracting the maximum firing rate from all data points in a curve and then taking the absolute values. The large number of units at cpd values of 0 and 100 are due to the fitting procedure which was constrained to means between 0 and 100 (see Methods).

A sizable fraction (27/49, 55%) of NCL neurons were significantly modulated during the sample phase. From visual inspection, many neurons seemed either tuned or anti-tuned to certain spatial frequencies (as e.g. in Figures 4A and 4BC, respectively). If NCL neurons code for reward-predicting stimuli, as has been suggested before [20], [23], the peaks of the neurometric tuning functions should be distributed closely to 16 cpd, the value of S+. To investigate this issue, we fitted neurometric gradients by Gaussian distributions (see Methods). This was done both for the original neurometric gradients as well as for their mirror image, obtained by flipping the gradients along the horizontal axis, in order to capture profiles such as those in Figure 4BC. The results can be seen in Figure 5 (left: regular gradients, right: inverted gradients). Most of the neurometric gradients which could be fitted reasonably well (r^2^>0.5, gray bars) had peaks (regular) or troughs (inverted) that were located in the vicinity of S+ (gray bars in Figure 5), and this finding was more pronounced for inverted gradients, i.e. those neurons which fired for stimuli dissimilar to S+ (as in Figure 4BC); medians cpds were 20.03 cpd and 18.97 cpd for regular and inverted gradients, respectively.

In a similar vein, 18 of 27 neurons with significant firing rate modulation during the sample phase additionally exhibited significant n-p correlations, most (15) of them negative. Conversely, only 4 of 22 neurons without significant firing rate modulation during the sample phase also showed significant n-p correlations (two positive, two negative). Of the 17 neurons with negative n-p correlations, 11 units showed reduced responding to S+, and 5 showed increased responding to one or both of the most extreme spatial frequencies. Thus, most of the negative n-p correlations are due to neurons exhibiting reduced responding to S+ (as in Figure 4C) rather than increased responding to extreme spatial frequencies (as in Figure 4B).

The fact that those neurons which exhibited tuning to specific spatial frequencies had their tuning peaks or troughs close to S+, as opposed to a uniform spatial frequency tuning distribution suggests that the NCL does not simply provide a representation of spatial frequencies, but that the spike responses to the stimuli may signal the reward value of certain stimuli (see discussion).

#### Motor-related responding

A previous study [20] reported that firing rates of some NCL neurons peaked around 70 ms prior to optical registration of beak opening, suggestive of premotor involvement. Correlations between neurometric and psychometric generalization gradients therefore could be due to motor modulation, with either enhanced or reduced firing relative to key pecking, instead of representing either the spatial frequency of sample stimuli or reward expectancy. To investigate this issue, we compared spike counts in the interval −200 to −100 ms to the interval −100 to 0 ms before the first key peck to the initialization stimulus. By that criterion, only 5/49 single units showed signs of premotor activity. Four of these increased their firing rate slightly during key pecking and one reduced its activity; the latter was the only neuron with a significant n-p correlation (of negative sign). Thus, premotor activity cannot explain the majority of instances in which NCL neurons are (anti-) tuned to S+.

The foregoing analysis was conducted to allow for a direct comparison to the previous report, and it classified a similarly small fraction of neurons as ‘premotor’ (them: 3/97, 3%; us: 5/49, 10%). However, this analysis is limited in that it can only detect changes in firing rate before registration of a key peck. Importantly, key pecking is a complex motor act resulting from the interplay of head, neck and possibly eye movements [31] that is accompanied by proprioceptive feedback. Therefore, we devised another analysis to more closely investigate the degree to which NCL neurons are modulated during key pecking. We constructed peri-peck time histograms (PPTHs), i.e. PSTHs triggered relative to each registered key peck. For 32/49 single units, there were enough spikes in the vicinity of key pecks to allow for statistical testing (at least 5 spikes per bin). This analysis is more liberal than the previous one in that it does not ask whether firing rate increases or decreases around key pecking relative to baseline, but whether the response profile during key pecking is modulated during key pecking with or without a net change in firing rate.

**Figure 6:**
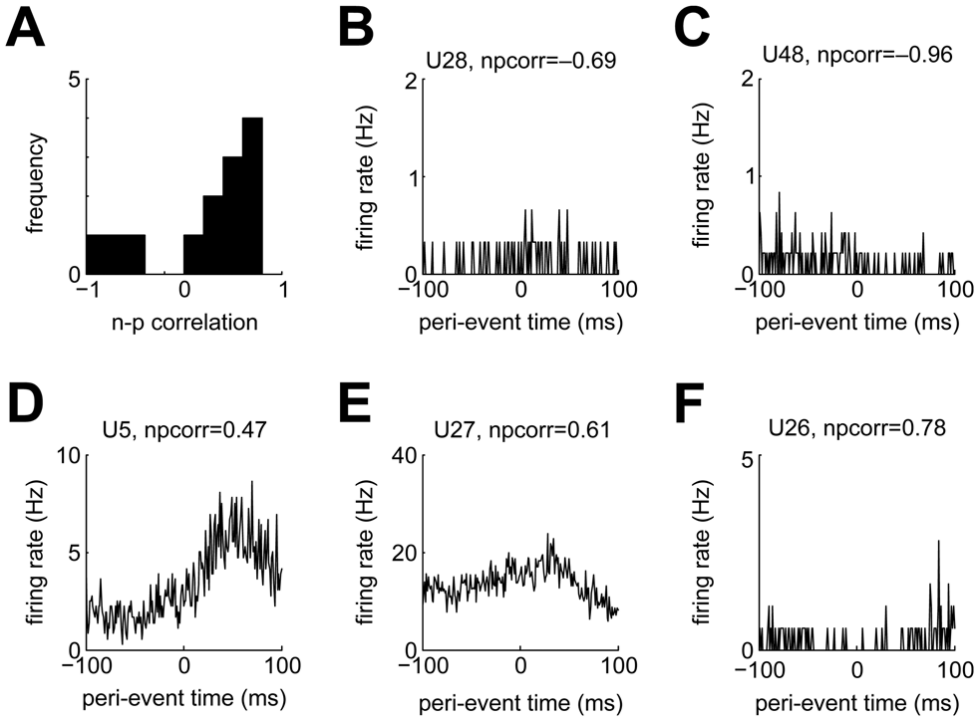
Motor modulation of NCL neurons. A) Distribution of n-p correlations for all neurons with significant motor modulation. B,C) PPTHs for the two neurons with significant negative n-p correlations. D,E) PPTHs for two example neurons showing increased firing ∼80 ms after key pecking. F) PPTH for an example unit with significant positive n-p correlation showing reduced firing around the time of key pecking.

In total, 13 of 32 (41%) tested neurons displayed significant firing rate modulation during key pecking. Most of these neurons (10/13) had positive n-p correlations (Figure 6A). There were two neurons with significantly negative n-p correlations which also showed significant motor-related modulation (these neurons' PPTHs are shown in Figures 6BC). However, both neurons' firing rate modulations cannot simply be described as inhibition: the neuron in Figure 6B rather shows a mild increase in response probability around the moment of the key peck, while the neuron in Figure 6C seems to fire somewhat stronger in the 100 ms before than in the 100 ms after the key peck. Figures 6DEF show PPTHs of the three neurons with both positive n-p correlations and significant motor modulation. The two neurons in Figure 6DE showed elevated firing some tens of milliseconds after key pecks. The other neurons with positive n-p correlations showed a reduction of firing rate during key pecking (Figure 6F). Two of the three putative interneurons were both significantly motor-modulated and exhibited positive n-p correlations (Figure 6DE).

For 10 neurons, we obtained enough data to additionally investigate the joint impact of the currently presented sample stimulus and key pecking (as in Figures 4AD, right panels). Of these, 7 neurons exhibited joint motor and sample modulation and could therefore code for contextual actions (such as key pecking during the presence of certain stimuli). Incidentally, all of these seven neurons showed positive n-p correlations.

### Neural activity during the outcome phase

#### Example neurons

**Figure 7:**
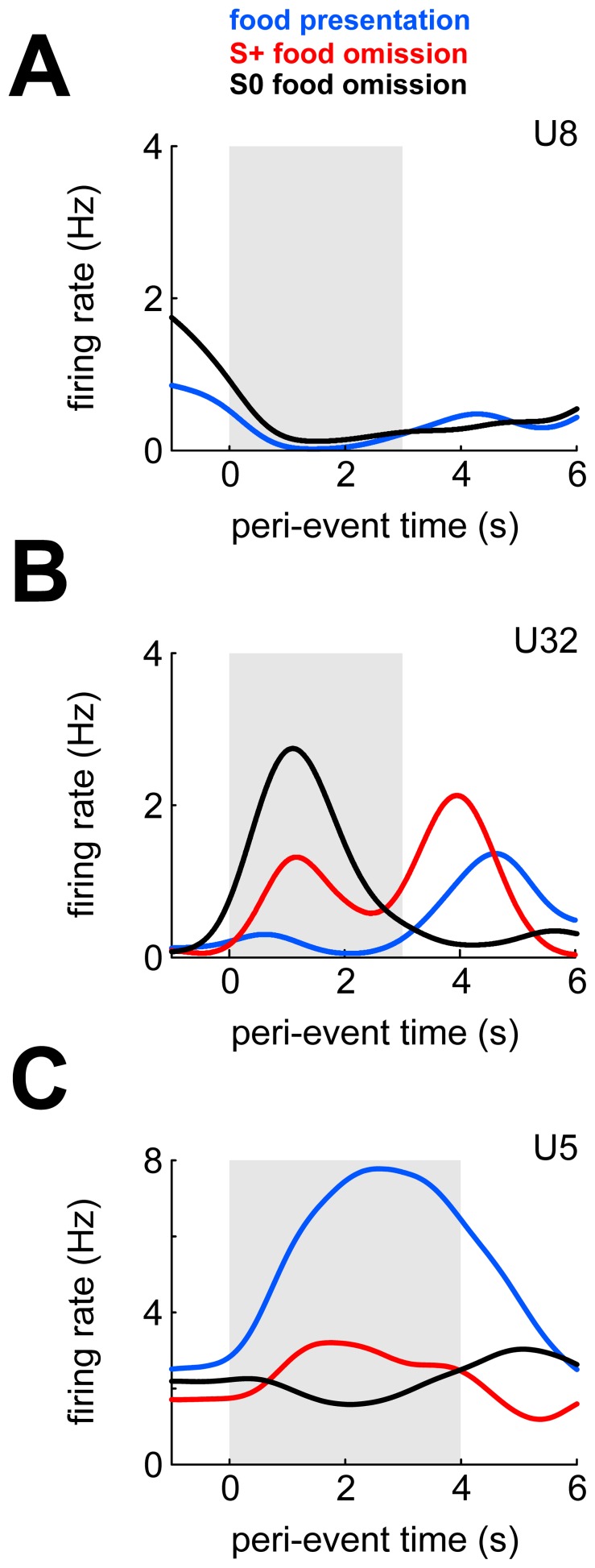
Response properties of three example neurons during the outcome phase. A) SDFs calculated during the outcome phase, shown separately for each type of outcome. Gray shaded area denotes duration of food hopper and feeder light operation on food trials and duration of feeder light operation on S+ food omission trials. Neither food hopper nor feeder light were activated on S0 food omission trials. B,C) As in A, but for different example neurons.

Figure 7 shows three example neurons' response profiles during the outcome phase. The neurons in Figure 7AB are the same neurons shown in Figure 4AB. The neuron shown in Figure 7A was inhibited when food was presented after a correct S+ response (p = 0.003) but not when food was omitted after an S0 response (p = 0.12; there were no food omissions after S+ responses in this session). The firing rate of the neuron in Figure 7B was not modulated during food presentation (p = 0.54), but increased immediately when food was omitted after either a correct S+ response (p = 0.06) or after an S0 response (p<10^−25^). Recall that during these latter events neither food hopper nor feeder light was operated (responses of unit 29 (Figure 4C) in the outcome phase were highly similarly to those of unit 32). Finally, the neuron in Figure 7C increased responding when reward was presented (p<10^−10^). This neuron's firing rate was not differentially modulated during the sample phase (χ^2^(11,309) = 8.8, p = 0.64; data not shown), but was strongly modulated during key pecking (χ^2^(3) = 461.8, p = 0; see Figure 6D).

#### Population response

Overall, 25/49 (51%) NCL neurons were significantly modulated during the outcome phase. During reward presentation, 8/25 (32%) neurons significantly increased and 15/25 (60%) neurons significantly decreased firing. When food was omitted after an S+ (S0) response, 8 (7) neurons increased and 5 (7) neurons decreased firing.

**Figure 8:**
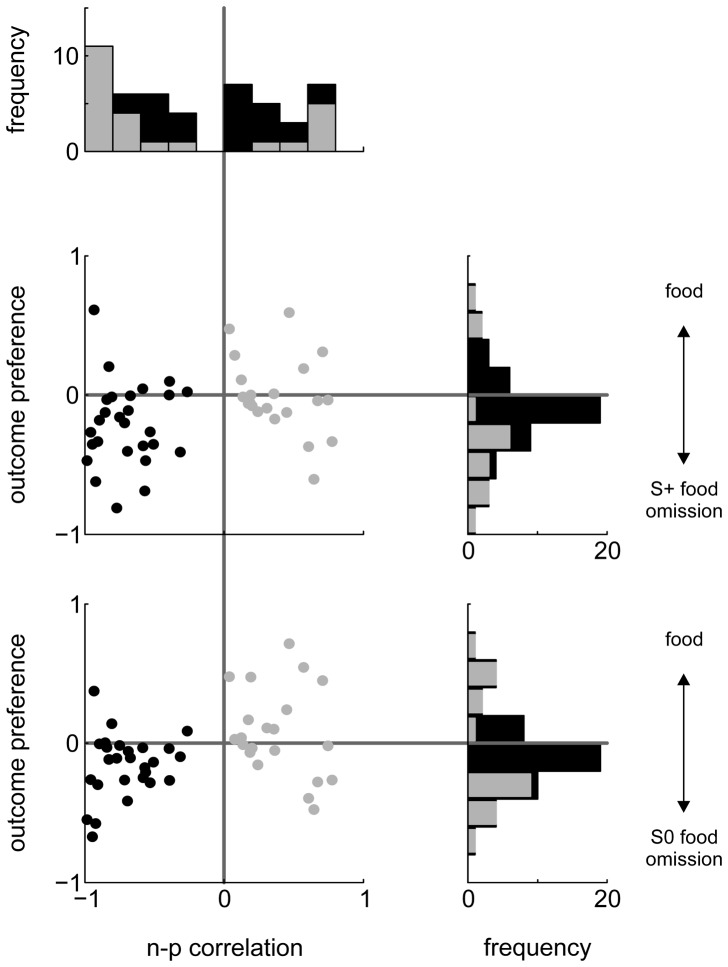
Relation of n-p correlation coefficients and outcome preference. Topmost panel: distribution of n-p correlations for all neurons (black bars) and for those with significant correlation (dark gray bars). The two scatterplots show the relation of n-p correlation values vs. outcome preference for food presentation vs. S+ and S0 food omission (middle and lowermost panels, respectively). Black dots denote neurons with negative n-p correlations; light gray dots denote neurons with positive n-p correlations. The vertical histograms to the right depict the distribution of outcome preferences for all neurons (black) and for those for which spike counts differed significantly (dark gray).

To obtain a direct comparison of NCL neurons' preference for rewarding and non-rewarding events, we contrasted responses to see which stimuli or events caused stronger firing rate elevations or reductions, and to what extent. To do this, we calculated the area under the receiver-operating characteristic curve (AUROC) for pairs of response distributions. AUROC varies between 0 and 1 and can be interpreted as the probability that an ideal observer could tell the two events apart by looking at spike counts alone. Following previous authors [44], we remapped the range to −1 and +1. We will refer to this measure as ‘outcome preference’. Outcome preference is coded such that neurons with values >0 fired more during reward presentation than during reward omission, while the converse is true for neurons with outcome preferences <0. Strikingly, only 3 neurons showed significantly stronger responding for food, while 14 neurons showed stronger responding for food omission after an S+ response (the results for food vs. food omission after an S0 response were 8 and 14; see histograms in Figure 8).

To sum up, many more NCL neurons increase firing to negative outcomes (omission of expected food reward) than to positive outcomes (food presentation), and more neurons were inhibited than excited by reward presentation, suggesting that negative outcomes have a greater impact on NCL neuronal activity than positive outcomes.

### Relationship of response patterns across both task phases

The foregoing analyses established that roughly half of all recorded NCL neurons were modulated in either phase of the behavioral task. In this section, we will explore to what extent neurons which are active in one phase are also active during the other phase.

We found that 15/49 (31%) neurons were significantly modulated during both phases and 12/49 (24%) during neither phase. Of the former 15 neurons, 9 exhibited significantly negative and 2 significantly positive n-p correlations. Figure 8 plots all neurons' n-p correlations against outcome preference for food vs. S+ and S0 food omission. Qualitatively, the neurons seem to fall into two groups, and this impression was confirmed by a cluster analysis which separated neurons with positive (gray) and negative (black) n-p correlations. Consistency of coding (i.e., firing for reward-predicting cues and for reward itself, or firing for cues predicting non-reward and to reward omission) should be visible by a preponderance of units in both the lower left (coding for negative events) and the upper right quadrants (coding for positive events). 21/49 neurons were located in the lower left quadrant and 7/49 in the upper right, and the overall distribution of data points differed significantly from that expected by chance (χ^2^(3) = 13.5, p = 0.004). Roughly the same results were obtained when repeating the analysis for a scatterplot of n-p correlation vs. relative preference for food or S0 food omission (χ^2^(3) = 15.8, p = 0.001).

Taken together, the above analyses demonstrate that NCL neurons which fire more for sample stimuli predicting a negative trial outcome also fire more for negative outcomes themselves. Instead, neurons with positive n-p correlations did not consistently fire when reward was presented.

### Histological reconstruction of recording sites

**Figure 9:**
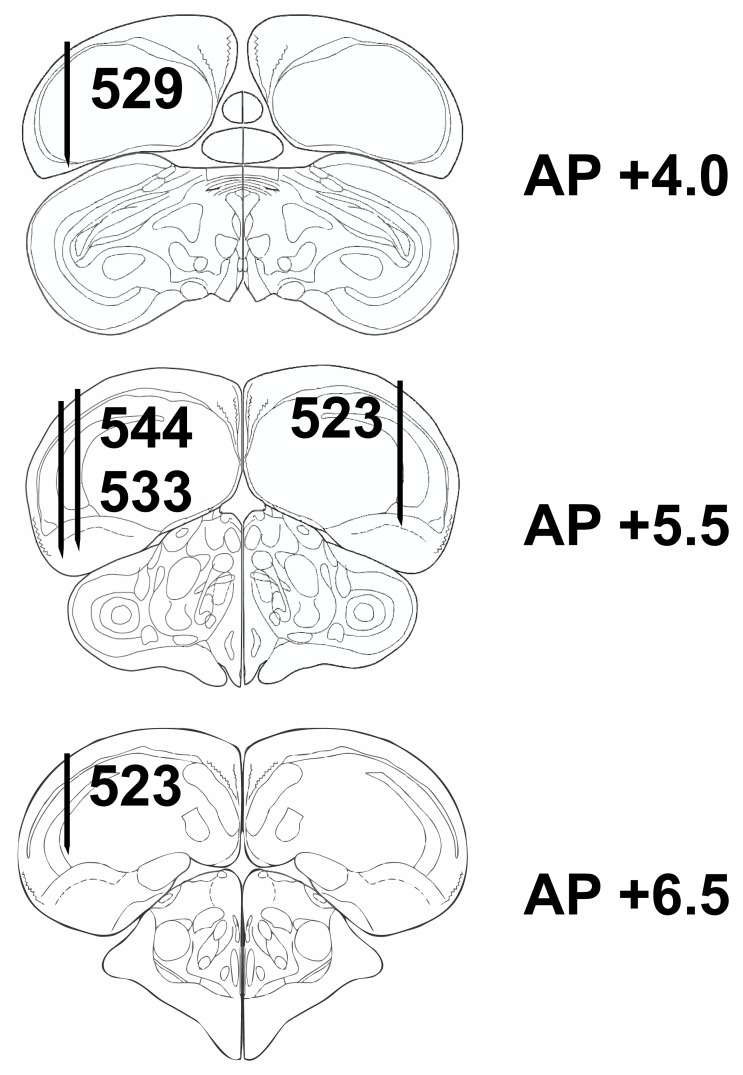
Histological reconstruction of the electrode tracks for four pigeons. Schematic sagittal sections of the pigeon brain, modified after the pigeon brain atlas of (Karten and Hodos, 2008). Black lines represent electrode tracks. Numbers next to electrode tracks identify individual animals. All tracks were within the boundaries of the NCL as defined in [8].

Figure 9 shows the histological reconstruction of recording sites which were all located within the borders of the NCL as defined by [8] for four of the animals; for the remaining animal, histological reconstruction was not possible. Qualitative inspection of the data did not reveal any obvious association of neuronal response properties (modulation in sample or outcome phase including n-p correlations and motor-related firing) and anatomical location.

## Discussion

We found that roughly two thirds of NCL neurons were significantly modulated during the behavioral task. NCL neurons responded to a variety of events, such as specific sample stimuli, sensorimotor- or proprioceptive events occurring during key pecking, and the quality of trial outcome (reward or non-reward).

There exist only a handful of studies which report NCL neural responses while the animals performed a behavioral task. In addition, most studies have employed working memory paradigms and reported results exclusively for that subset of neurons whose responses were modulated during delay phases [18], [46]–[48]. Our task lacks a working memory component, so our findings cannot directly be compared to those reports. Only two previous studies have analyzed neural responses in all trial phases as we did [20], [23], and their findings are fully consistent with our results. Both studies reported that NCL neurons respond to stimuli predicting reward and/or to stimuli predicting non-reward. In addition, both studies reported that some neurons responded to reward itself, although neither analyzed responses during reward omission.

### Do some NCL neurons code for reward expectancy or positive events?

Most previous authors have related NCL neural activity to reward expectancy [21], [47]–[49]. Indeed, in this as well as in previous studies [20], [23], [49], a subset of NCL neurons fired in response to cues predicting reward, and a few neurons responded during reward presentation.

The psychometric generalization gradient provides an index of reward expectancy [35]–[37]. Neurons which represent reward expectancy should, therefore, increase firing to sample stimuli to which the animal responded most vigorously, and this should be reflected in significant n-p correlations. It is tempting to assume that NCL neurons with positive n-p correlation represent reward expectancy; however, the evidence for this claim is weak. We found only five neurons with significantly positive n-p correlations, and for three of these, the positive n-p correlation could in principle be explained by enhanced firing during key pecking. Also, there was not a single unit exhibiting both a significantly positive n-p correlation as well as increased responding to reward itself. Accordingly, NCL neurons do neither seem to provide a reward prediction error of the kind found e.g. in midbrain dopamine neurons and prefrontal cortex [50], [51], nor do they seem to code for positive events per se [30], [52]. This again is in line with previous studies: while both [20], [23] found NCL neurons responding to reward-predicting stimuli as well as to liquid reward, neither found neurons which consistently fired for both kinds of positive events (reward-predicting stimuli and reward itself).

### Do some NCL neurons code for non-reward or negative events in general?

About a third of all NCL neurons exhibited significantly negative n-p correlations. In principle, negative n-p correlations could arise from an inhibition of firing during key pecking. However, for the vast majority of neurons with negative n-p correlations, there was no evidence of motor-related firing rate inhibition that could give rise to the observed negative n-p correlations. Additionally, many neurons showed elevated firing to S0s with extreme spatial frequencies instead of or in addition to suppressed responding to S+ (see Figure 4B), which cannot be explained by a simple sensorimotor account.

It could be argued that NCL neurons simply responded to specific spatial frequencies regardless of their reward value. However, we hold this interpretation unlikely for several reasons. Firstly, virtually all neurons with significant modulation during the sample phase were tuned (or anti-tuned) to S+, which would be surprising for a set of purely visual neurons. Secondly, these neurons' response pattern during the sample phase was predictive of their response pattern during the outcome phase in which the sample stimuli were absent: many of these neurons were inhibited during reward presentation but excited when food was omitted after either a response to S+ or an S0. The latter two conditions again differed in their sensory properties because the feeder light was on during S+ food omission but off during S0 food omission. To sum up, this set of neurons fires a) for low- and high- but not medium-frequency sample stimuli, b) when food is omitted after S+ (feeder light on), and c) when food is omitted after S0 (feeder light off). This conjunction cannot be explained by pointing to spatial frequency selectivity but makes perfect sense in the framework of neurons coding a negative reward prediction error as e.g. neurons in primate anterior cingulate cortex [53] and the lateral habenula [54], or alternatively neurons coding for negative events per se (as has been suggested for amygdala neurons [30]).

### Motor modulation

This is the first study to investigate the conflation of sensory and motor signals on single NCL units. Conducting a more sensitive analysis than previous studies [20], [23], we found that that the NCL contains more sensorimotor neurons than previously thought. Accordingly, it will be important in future studies to tightly register more aspects of motor output than merely key pecking, for example head movements. It will be difficult to disentangle whether motor-related modulation of NCL neurons is indicative of (pre-) motor output, exteroceptive or proprioceptive input, because key pecks result from a complex interplay of head and body movements [31], [45]. There exist prominent bidirectional projections from NCL to both sensorimotor striatum and the somatomotor part of the arcopallium [8] which provide possible sources of sensorimotor input to NCL. The convergence of action- and stimulus-related information in NCL neurons constitutes a similarity to (rodent) orbitofrontal cortex [44] and thus further supports the notion that the NCL is functionally equivalent to prefrontal cortex [9], [22]. Incidentally, orbitofrontal cortex also contains a preponderance of neurons responding to reward omission compared to reward presentation [42].

### Conclusions

Our present results demonstrate that NCL neurons show highly diverse response profiles related to stimuli, response execution, and action outcomes. The NCL is well situated to communicate its output to brain regions involved in reward processing and action planning, such as both limbic and sensorimotor striatum and arcopallial motor fields [8]. The neurons hypothesized to represent a negative value signal could form part of an evaluation circuit dedicated to optimize behavior in the face of both rewarding and aversive events [55]. Future studies could probe NCL neurons with a wider array of negative events as realized here, including punishment. In addition, it will be important to tightly register the subjects' motor output during all phases of the task to avoid confounding cognitive signals such as reward expectancy with motor-related signals.
